# Analysis of Runs of Homozygosity in Aberdeen Angus Cattle

**DOI:** 10.3390/ani14152153

**Published:** 2024-07-24

**Authors:** Vladimir Kolpakov, Alexey Ruchay, Dianna Kosyan, Elena Bukareva

**Affiliations:** 1 Federal Research Centre of Biological Systems and Agro-Technologies of the Russian Academy of Sciences, 460000 Orenburg, Russia; vkolpakov056@yandex.ru (V.K.); kosyan.diana@mail.ru (D.K.); elenka_rs@mail.ru (E.B.); 2 Department of Biotechnology of Animal Raw Materials and Aquaculture, Orenburg State University, 460000 Orenburg, Russia; 3 Department of Information Security, South Ural State University, 454080 Chelyabinsk, Russia; 4 Department of Mathematics, Chelyabinsk State University, 454001 Chelyabinsk, Russia

**Keywords:** cattle, Aberdeen Angus breed, genomic architecture, loci under pressure, genome-wide associative studies

## Abstract

**Simple Summary:**

Simple Summary: This article studies runs of homozygosity (ROHs) across the genome using high-density single-nucleotide polymorphism (SNP) arrays for the structural and functional annotation of genes localized inside or in close proximity to selected sections of the genome of Aberdeen Angus cattle. The obtained results could serve as a foundation for future studies aimed at identifying the genes and markers required to determine the most significant productive traits of cattle. The data confirm the effect of targeted selection on increasing the height, body size, and live weight in the population of Aberdeen Angus cattle.

**Abstract:**

A large number of cattle breeds have marked phenotypic differences. They are valuable models for studying genome evolution. ROH analysis can facilitate the discovery of genomic regions that may explain phenotypic differences between breeds affecting traits of economic importance. This paper investigates genome-wide ROH of 189 Aberdeen Angus bulls using the Illumina Bovine GGP HD Beadchip150K to structurally and functionally annotate genes located within or in close ROH of the Aberdeen Angus cattle genome. The method of sequential SNP detection was used to determine the ROH. Based on this parameter, two ROH classes were allocated. The total length of all ROH islands was 11,493 Mb. As a result of studying the genomic architecture of the experimental population of Aberdeen Angus bulls, nine ROH islands and 255 SNPs were identified. Thirteen of these overlapped with regions bearing ‘selection imprints’ previously identified in other breeds of cattle, and five of these regions were identified in other Aberdeen Angus populations. The total length of the ROH islands was 11,493 Mb. The size of individual islands ranged from 0.038 to 1.812 Mb. Structural annotation showed the presence of 87 genes within the identified ROH islets.

## 1. Introduction

The use of single-nucleotide polymorphism (SNP) markers [1] to assess the productivity of cattle is becoming increasingly necessary in commercial animal husbandry [2,3], and thanks to the presence of panels containing thousands of different SNPs, it has become possible to predict the breeding value, to study the effects of inbreeding at the genomic level, and to assess the probabilities of autozygosity. To accomplish this, it was proposed to study the structure of runs of homozygosity (ROHs) as a possible approach for measuring the level of inbreeding in animal husbandry [4,5].

ROHs are continuous homozygous segments in the genome. Being an important part of the genome of an organism, these patterns represent an important source of information for studying the structure of the genome. It is assumed that ROHs can carry harmful recessive mutations and, therefore, can lead to inbreeding depression. For this purpose, it is necessary to estimate their length. They are used as a temporary indicator of inbreeding events; long ROHs are most likely formed as a result of recent inbreeding [6], while short ones, on the contrary, suggest more distant inbreeding. It is assumed that the occurrence of ROHs may be the result of both artificial and natural selection [7]. Based on the theory of genetic cleansing, it is assumed that ancient inbreeding leads to less adverse effects than more recent inbreeding, which may indicate more serious adverse consequences [8]. The study of ROH has formed the basis of a large number of genetic programs that promote genetic progress. According to recent studies, such programs are being implemented in many breeds, including South African Bonsmara, Hereford, and Afrikaner, Nguni breed, Belgian blue, and others [9,10,11].

Since a large number of cattle breeds have marked phenotypic differences and, therefore, represent valuable models for studying the evolution of the genome during breeding and domestication, ROH analysis can contribute to the detection of genomic regions that could explain the phenotypic differences between the breeds that affect the traits that have economic importance. Previously, the structure of ROH in a large number of cattle breeds has been studied, especially in the dairy industry.

Aberdeen Angus cattle have rarely been the focus of such studies, but recently, their value as a possible alternative to intensively farmed breeds has become apparent, especially because of their greater adaptability to environmental changes and the potential for living in less productive areas [12,13]. This can be attributed to the expansion of the Aberdeen Angus cattle breed in Russia. According to the data for 2021, the number of Aberdeen Angus cattle rose from 2000 to 100,000 [14]. Aberdeen Angus cattle are distinguished by a high growth rate, good indicators of fertility and life expectancy, pronounced meat qualities, and individual adaptability to environmental conditions [15,16]. As with many other breeds, genomic information about Aberdeen Angus has become available only recently, after routine genotyping. This information makes it possible to identify and describe genes and functional pathways involved in the genomic architecture of traits of economic or functional interest [17,18]. This breed represents an untapped source of diversity for the livestock sector and an opportunity to conduct ROH analysis on key markers that have not been investigated.

The objective of this work was to analyze SNP marker genotyping data based on the analysis of homozygosity patterns (ROH islands) and to perform structural and functional annotation of genes located within or in close proximity to the analyzed regions of the bovine genome.

## 2. Materials and Methods

### 2.1. Ethical Statement

All experimental procedures and ethical standards were approved by the Committee for Control over the Maintenance and Use of Laboratory Animals in accordance with the “Policy of the Scientific Center for Work with Laboratory Animals” of the Federal State Budgetary Scientific Institution “Federal Research Centre of Biological Systems and Agrotechnologies of the Russian academy of sciences”. Extracts from the minutes of the meeting of the Commission for Control over the Maintenance and Use of Laboratory Animals No. 4, dated 17 January 2021, were received on 20 March 2022. During the research, measures were taken to ensure a minimum of animal suffering and to reduce the number of experimental samples studied.

### 2.2. Animals and Genotyping

Bulls of the Aberdeen Angus breed (*n* = 189) were selected for this study. The subjects consisted of fattening animals. Animals were not half-siblings and were derived from crosses between representatives of different Aberdeen Angus subtypes. At the time of the experiment, the average age of the animals was 17.4 ± 0.04 months, and their average body weight was 621.79 ± 1.84 kg. The animals were kept in a separate pen at a feedlot located in the Ramonsky district of the Voronezh Region, Russia. All the animals were male and of the same age. The feeding and maintenance conditions were identical for all animals.

Genomic DNA was extracted from whole blood using “DNA-Extran” (Syntol LLC, Moscow, Russia). The concentration of genomic DNA was detected by Qubit (Thermofisher, Waltham, MA, USA), and the quantity of DNA was estimated by Nanodrop ND-1000 (ThermoFisher, Waltham, MA, USA). The 189 animals were genotyped with the Illumina Bovine GGP HD Beadchip150K (Illumina, Inc., San Diego, CA, USA) (HD; 138,974 markers) following standard procedures of the manufacturer.

Quality control and the filtering of genotyping data for each SNP and each sample was performed using the PLINK 1.9 software package (http://zzz.bwh.harvard.edu/plink/ (accessed on 18 July 2024)) using the following filters (the corresponding commands in the PLINK program are given in parentheses): a call-rate for all studied SNPs for an individual sample not lower than 90% (--mind); a call-rate for each of the studied SNPs for all genotyped samples of at least 90% (--geno); the deviation of SNP genotypes from the Hardy–Weinberg distribution in the aggregate of tested samples with a confidence of *p*-value < 10^−6^ (--hwe).

### 2.3. ROH Detection and Classification

To determine the ROH, a sequential SNP detection method was used, implemented using the detect RUNS R package 4.4.1 [19]. To exclude the underestimation of the number of ROHs longer than 8 Mb, the presence of one SNP with a missing genotype and no more than one possible heterozygous genotype was assumed [20]. Due to the presence of strong no-equilibrium coupling at a distance of up to 100 kb [21], we set the threshold of the minimum length for one ROH at 500 kb. To minimize false positive results, we calculated the minimum number of SNPs (*l*) as proposed by Links et al. [22] and later modified by Purfield et al. [23]:

(1) $$l=\frac{\log_{e}\frac{\alpha }{n_{s}\times n_{i}}}{\log_{e}\left(1-\bar{het}\right)},$$

where *n_s_* is the number of genotyped SNPs per individual, *n_i_* is the number of genotyped individuals, *α* is the percentage of false positive ROHs (in our study, it was set at 0.05) and $\;\bar{het}$ is the average heterozygosity calculated for all SNPs. According to the calculations, the minimum number of SNPs was 20.

As a threshold for the minimum proportion of animals within the breed that have overlapping ROHs, we selected the value of 50%, as proposed by a number of authors [24,25,26]. The threshold for the minimum length and number of SNPs in overlapping segments was not set.

### 2.4. Detection of Common ROH and Gene Annotation

Multiple linear regression analysis implemented in Plink 1.90 was used to identify associations of ROH and phenotype (withers and rump height). The Bonferroni null hypothesis test with a threshold value of *p* < 1.5 × 10^−6^ was used to confirm the reliable influence of ROH and to identify significant regions in the genome. Data visualization was carried out in the qqqman package using the R programming language [27].

Clustering of the functional annotation was carried out in the DAVID web program 2024q1 (Functional Annotation Chart) (https://david.ncifcrf.gov/ (accessed on 18 July 2024)), and the enrichment coefficient used in the calculations was –log_10_(*p*) > 1.3, which corresponds to *p* < 0.05.

## 3. Results

### 3.1. Identification and Distribution of ROH Islands

**Figure 1:**
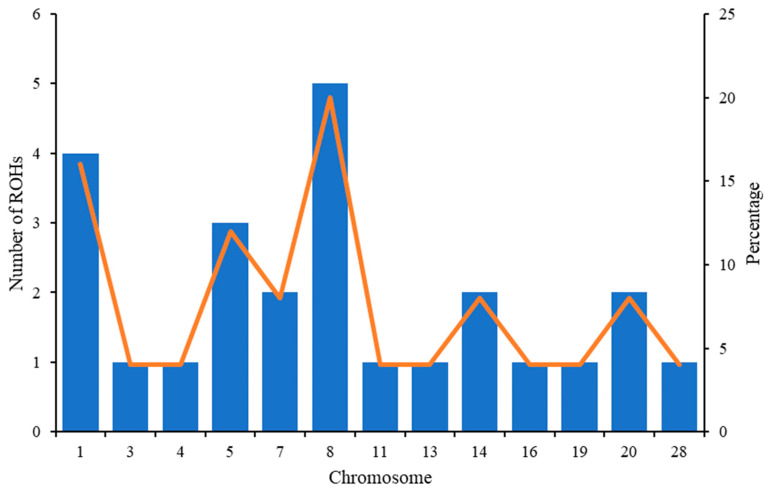
The number of ROHs and the percentage coverage per chromosome in Aberdeen Angus breed animals. The vertical bars show the total number of ROHs per chromosome and the line shows the percentage of chromosome covered with ROHs.

The data showed that in the genome of the experimental population of Aberdeen Angus cattle (n = 189), there are 25 islands of ROHs, which are found in the genome of more than 50% of the animals. The distribution of the amount of ROHs between chromosomes and their percentage of chromosomes is shown in Figure 1.

In addition to estimating the number of ROH islands, special attention should be paid to the length of these sections. Based on this parameter, two ROH classes were allocated. The total length of all ROH islands was 11,493 Mb. Table 1 presents the main statistical parameters for each of the classes. The average length of the sections is 0.460 Mb, and the main coverage of the genome is provided by segments ranging in size from 0.1 to 0.5 Mb. The size of individual islands varied from 0.038 to 1.812 Mb.

**Figure 2:**
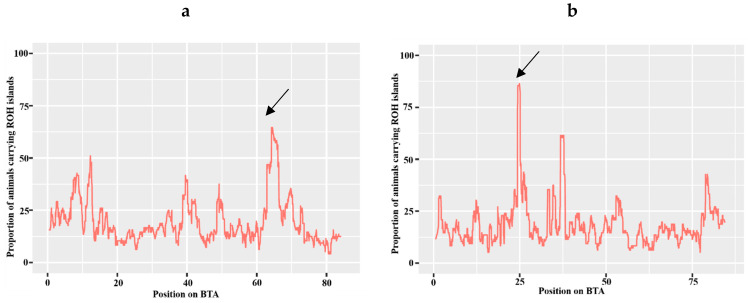
Distribution of ROH islands in the experimental group of Aberdeen Angus breed: (**a**) on BTA13; (**b**) on BTA14. Arrows indicate islands that occur in the majority of animals in the study.

The longest ROH island was identified on BTA13 (Figure 2a), and an increase in the threshold to 70% showed the presence of one 0.772 Mb ROH island, localized on BTA14. This island was detected in the genome of more than 85% of the animals (Figure 2b).

The structural annotation showed the presence of 87 genes inside and distributed among 17 different ROH islands. The initial and final position of ROH, its length, the number of SNPs, and the number of genes in a particular chromosome are shown in Table 2. In order to assess the potential functional significance of the ROH islands, the quantitative content of genes in the identified regions was analyzed.

### 3.2. Carrying Out Structural Annotation of Genes Localized inside or in Close Proximity to Selected Sections of the Animal Genome

Table 3 summarizes comparative studies of candidate positional genes localized inside the ROH islands in the sample of Aberdeen Angus cattle with the results of a meta-analysis performed by Randhawa et al. [12,28,29] based on the analysis of 56 GWAS, including 70,743 animals of 90 breeds and crossbreeds.

Table 3 shows that out of the 25 identified ROHs, 13 overlap with regions bearing “selection imprints” identified earlier in other livestock breeds, of which five regions were identified in other populations of the Aberdeen Angus breed. Areas were found in BTA1 and BTA8, which are characteristic of Holstein and Simmental breeds; in BTA1, BTA4, BTA7, and BTA11 of limousines; BTA4, BTA7, BTA8, BTA14, and BTA20 of Belgian blue; and BTA4, BTA7, BTA8, BTA13, and BTA14 of Aberdeen Angus.

### 3.3. Carrying Out Functional Annotation of Genes Localized inside or in the Immediate Vicinity of Selected Sections of the Animal Genome

Functional annotation of 87 genes localized within the identified regions showed the presence of 84 genes with known functions described in terms of gene ontology. The analysis of the results of the functional annotation of this list of genes revealed the presence of only one reliable annotated cluster with an enrichment coefficient –log_10_(*p*) > 1.3, which corresponds to *p* < 0.05 (Table 4).

This cluster includes nine genes involved in the processes of RNA transformation. Five of these genes, including *HNRNPA2B1*, *PURA*, *RBM12*, *RBM39*, and *RPS20*, are located in ROH islands that overlap with regions identified by other authors as being under selection pressure in animals of the Aberdeen Angus breed (see Table 3).

## 4. Discussion

The study of the genomic architecture of different cattle breeds has been made possible by the development of Genome-Wide Association Studies (GWAS), which can identify genetic regions for phenotype development. Annotating these loci (ROHs) as genes and their functional parts that contain significant SNPs is an important step in the GWAS process to perform linkage to the phenotype under test [30,31]. Characterizing the distribution and length of ROHs in a population can help reveal its evolutionary history, inappropriate mating patterns that lead to increased inbreeding, and identify close genomic associations with phenotypic traits. For example, animals with high ROH (with long segments) may be excluded from mating schemes or assigned a lower priority to minimize loss of genetic diversity and maintain or increase effective population size [32,33,34,35]. This is especially important for the study of productive qualities of beef cattle, in particular Angus cattle.

Recently, the quality of their meat has improved due to abundant marbling caused by deposits of intramuscular fat. This was achieved through the intensive use of several excellent sires with high evaluation of breeding value in terms of marbling. This rapid improvement led to a decrease in effective population size and an increase in inbreeding. Most producers still have enough questions about the acceptable level of inbreeding on their farms to continue genetic progress but not cause any negative reduction in productivity, fertility, or health indicators. Inbreeding itself is not a factor that should influence selection decisions. It affects the profitability of the animal as well as the decision to pair to focus on genetic progress and ultimately profitability.

In recent years, studies aimed at detecting positive selection using ROH signaling have also been conducted on cattle [36,37,38]. In our study, the suitability of ROH islands for scanning selection signatures was confirmed due to several matches with QTLs of traits under selection and previously reported selection signatures of beef cattle. Results showed that of the 25 ROHs identified, 13 overlapped with regions carrying “selection imprints” identified previously in other cattle breeds, of which five regions were identified in other populations of the Aberdeen Angus breed. Given that ROH islands result from selection pressure, where only individuals with the desired phenotype are selected for breeding—and thus certain combinations of genotypes are subjected to indirect selection—the varying proportion of ROHs in the genome is likely the result of the different selection strategies used in sorting each breed. In addition, regions on BTA4, BTA7, BTA8, BTA13, and BTA14 were found near genes responsible for muscle development, double muscle, meat tenderness, and intramuscular fat content [39]. The detected ROH islands indicate common selection events at BTA13 and BTA14, in genomic regions containing genes known to be associated with growth in mammals [40,41,42], and thus presumably affecting growth traits undergoing selection in the Angus population. These ROHs have previously been reported as selection traits in Angus [43] and were significantly associated with Brahman body mass [44] in a study based on the reference UMD3.1 as part of the work of the Bovine Genome Sequencing and Analysis Consortium. Candidate genes for BTA13 include the *GDF5* gene, which belongs to a family of bone morphogenetic proteins that stimulate bone formation and regulate growth [45], and *MHY7B*, which is expressed in skeletal muscle and repressed in double-muscled breeds [46]. The BTA14 region including *PLAG1* and *XKR4* genes is well known for its pleiotropic effects on economically important traits of beef cattle, such as rump thickness, rib area, body and carcass weights [47,48,49], as well as on serum levels of growth hormones, such as *IGF1*, as well as on serum levels of growth-related hormones [50]. The BTA8 region is represented by a wide range of traits, including pregnancy rate, stillbirth rate, sperm motility, net worth, and milking rate in Brangus cattle [51]. Studies in Angus, Brahman, and Angus–Brahman populations have identified QTLs for pregnancy rate, male fertility, and intramuscular fat in this specific genomic region [52,53,54,55]. The BTA8 gene is represented by a wide range of traits including daughter pregnancy rate, stillbirth, sperm motility, net value, and milking rate in Brangus cattle [51]. Studies involving Angus, Brahman, and Angus–Brahman crossbreed populations have identified QTLs for daughter pregnancy rate, male fertility, and intramuscular fat in this specific genomic region [52,53,54,55]. The BTA7 region comprising SNP rs110428791, which is an intronic variant of gene *UNC5A* mapped at this selection signature, was previously associated with reproductive performance in taurine breeds, in a study based on the reference genome UMD3.1

In addition, the homologous genes *RNF44* and *UIMC1* have been associated with reproductive risks and reproductive life expectancy in women [56,57]. These data suggest selection pressure in an Angus population with a balance between growth and reproductive traits. In addition, this genomic region was previously identified as a QTL associated with resistance to viral load in Holstein cattle [58] and includes genes that have shown differential expression between susceptible and resistant hosts to the mite (*KIF3A*) [59], mangal-infected and uninfected animals (IL4, IL5, and IL13) [60], and healthy and mastitis-affected cows (IL4) [61]. In addition, this selection trait was associated with the adaptation of taurine breeds to harsh environmental conditions [62], and of indicine breeds to less harsh but still difficult environmental conditions [63].

The analysis of the genetic structure of the cattle population on monogenic hereditary diseases is an urgent problem, the solution of which will allow us to carry out targeted control over the spread of mutations. This will make it possible to increase the resistance of breeding stock and safety of breeding stock, exclude the importation of bulls carrying genetic cargo, ensure the introduction of healthy animals into breeding herds, and adjust breeding programs. Among specific loci, quantitative trait loci and disease resistance loci are the most intensively studied. In this regard, *CD18, UMPS*, and *SLC35A3* genes, point mutations which are associated with diseases such as BLAD, DUMPS, and CVM, have been widely investigated.

The congenital immunodeficiency syndrome (BLAD) is caused by a point mutation in the coding part of the autosomal *CD18* gene and controlling the synthesis of the glycoprotein β-integrin. Damage to the structure of this protein leads to multiple defects in leukocyte function. In the homozygous recessive state, it is lethal; the immune system of animals is unable to resist viral and bacterial infections, which leads to their death at an early age [64,65,66]. DUMPS (Deficiency of Uridine Monophosphate Synthase) is a monogenic autosomal recessive disease due to a metabolic disorder due to deficiency of the enzyme uridine monophosphate synthetase associated with the reproductive function of animals, and it affects the survival of offspring, causing embryo death, usually after the first 40 days of development, that is, at the stage of implantation of the embryo in the uterus [67,68,69]. Complex spinal deformity syndrome (CVM) is caused by a recessive mutation associated with the *SLC35A3* gene. Characteristic features of CVM carriers are general underdevelopment, shortened neck, fused and deformed vertebrae, scoliosis, rib malformations, and deformed joints of fore and hind limbs [70,71].

Thus, the study found SNPs that are associated with important economic and utility traits. In addition, ROH analysis in Aberdeen Angus cattle showed the presence of relatedness between Angus and other breeds. ROH parameters, in general and calculated by length class, reflect the effects of actual population size and selective history. As a result, genomic information and genealogical data play a critical role in facilitating the management of small populations, with the primary goal of planning the right pairs for mating, reducing inbreeding, and increasing the productivity of specific production traits.

## 5. Conclusions

The study of the genomic architecture of a population of Aberdeen Angus bulls, based on the analysis of 106,828 SNPs that passed quality control, found 25 ROH residues in the genome of more than 50% of the animals. The total length of the ROH islands was 11,493 Mb. The size of individual islands ranged from 0.038 to 1.812 Mb. Of the 25 regions identified, 13 overlap with regions bearing “selection imprints” identified earlier in other cattle breeds, of which five regions were identified in other populations of the Aberdeen Angus breed.

The structural annotation showed the presence of 87 genes inside the identified ROH islands, which were distributed among 17 different ROH islands. The functional abstract showed the presence of 84 genes with known functions described in terms of gene ontology. Among the ROH islands, a region was found on BTA14, including the genes *XKR4*, *TMEM68*, *TGS1*, *LYN*, *RPS20*, *MOS*, *PLAG1*, and *CHCHD7*, which overlapped with the well-known “selection imprints” in the genome of cattle of a number of breeds (Aberdeen Angus, Hereford, Limousine, Charolais, Belgian Golden, Simmental, Holstein, Yaroslavl, and Kholmogory). The *PLAG1-CHCHD7* region in many cattle breeds is a strong candidate gene responsible for growth and body size, including height. The data obtained confirm the effect of targeted selection on increasing the height, body size, and live weight in Aberdeen Angus cattle.

This work is a continuation of a series of studies conducted under research project No. 21-76-20014 [12]. The data obtained require refinement on a larger sample; they expand our understanding of the assessment of the effect of artificial selection on genome variability and will be useful for the development of a system of marker-based genomic selection of Aberdeen Angus cattle.

## Figures and Tables

**Table 1:** Distribution of ROHs of different lengths.

ROH Length (Mb)	ROH (n)	Percent (%)	Mean Length (Mb)	SD	Min	Max
ROH 0.5–1	8	32	0.709	0.167	0.508	0.974
ROH 1–2	1	4	1.812	1.812	1.812	1.812
Total ROHs	25	100	0.460	0.392	0.038	1.812

**Table 2:** List of genomic regions of extended homozygosity detected in the experimental group of Aberdeen Angus bulls.

Chromosome	Start (bp)	End (bp)	Length (bp)	SNPs	Genes
7	52,757,805	53,310,739	552,934	16	8
8	55,718,826	56,283,438	564,612	23	1
8	93,132,174	93,792,855	660,681	20	1
11	52,843,813	53,351,806	507,993	18	-
13	64,228,423	66,040,326	1,811,903	47	34
14	24,326,513	25,098,364	771,851	57	8
14	36,817,690	37,791,273	973,583	23	-
20	30,865,802	31,752,546	886,744	29	8
28	2,116,842	2,869,287	752,445	22	1

**Table 3:** The results of comparative studies of candidate positional genes localized inside the identified ROH islands with the results of a meta-analysis performed by Randhawa et al. [29].

BTA	Position (Mb)	Genes	Breed
7	52.76–53.31	*NRG2*, *PURA*, *IGIP*, *CYSTM1*, *PFDN1*, *HBEGF*, *SLC4A9*, *EIF4*, *EBP3*	Aberdeen Angus, Charolais, Limousine, Belgian Blue,
8	55.72–56.28	*TLE4*	Belgian Blue
	90.98–91.40	*SPIN1*	Holsteins
	93.13–93.79	*GRIN3A*	Aberdeen Angus
13	64.23–66.04	*ASIP*, *AHCY*, *ITCH*, *DYNLRB1*, *MAP1LC3A*, *PIGU*, *TP53INP2*, *NCOA6*, *GGT7*, *ACSS2*, *GSS*, *MYH7B*, *bta-mir-499*, *TRPC4AP*, *EDEM2*, *PROCR*, *MMP24*, *EIF6*, *FAM83C*, *UQCC1*, *GDF5*, *CEP250*, *ERGIC3*, *SPAG4*, *CPNE1*, *RBM12*, *NFS1*, *ROMO1*, *RBM39*, *PHF20*, *SCAND1*, *CNBD2*, *EPB41L1*, *AAR2*	Aberdeen Angus
14	24.33–25.10	*XKR4*, *TMEM68*, *TGS1*	Belgian Blue, Simmentals, Limousine, Holsteins, Charolais
		*LYN*, *RPS20*, *MOS*, *PLAG1*, *CHCHD7*	Aberdeen Angus, Belgian Blue, Simmentals, Limousine, Holsteins, Charolais
		*PLAG1*, *CHCHD7*	Aberdeen Angus, Herefords, Belgian Blue, Simmentals, Limousine, Holsteins, Charolais
20	5.39–5.79	*NSG2*	Belgian Blue
20	30.87–31.75	*NNT*, *PAIP1*, *C20H5orf34*, *TMEM267*, *CCL28*, *HMGCS1*, *NIM1K*, *ZNF131*	Holsteins

**Table 4:** The results of enriching the lists of genes specific to the population of young Aberdeen Angus cattle.

Cluster	Category	Terms	*p*	Genes
Cluster 1, enrichment coefficient = 1.52	UP_SEQ_FEATURE	DOMAIN: RRM	0.012	*RBM39*, *SYNJ1*, *HNRNPA2B1*, *RBM12*, *CPEB4*
	INTERPRO	IPR000504: domain of the recognition motive of RNA	0.013	
		IPR012677: nucleotide binding, alpha-beta harness	0.022	
	GOTERM_MF_ DIRECT	GO:0003723~RNA binding	0.018	*PURA*, *RBM39*, *EYA1*, *SYNJ1*, *RPS20*, *PAIP1*, *HNRNPA2B1*, *RBM12*

## Data Availability

The data presented in this study are available.
